# HTLV-1 as a Model for Virus and Host Coordinated Immunoediting

**DOI:** 10.3389/fimmu.2019.02259

**Published:** 2019-09-24

**Authors:** Talia M. Mota, R. Brad Jones

**Affiliations:** ^1^Infectious Diseases Division, Department of Medicine, Weill Cornell Medical College, New York, NY, United States; ^2^Program in Immunology and Microbial Pathogenesis, Weill Cornell Graduate School of Medical Sciences, New York, NY, United States

**Keywords:** immunoediting, HTLV-1, HIV, viral reservoir, ATL (adult T-cell leukemia)

## Abstract

Immunoediting is a process that occurs in cancer, whereby the immune system acts to initially repress, and subsequently promote the outgrowth of tumor cells through the stages of elimination, equilibrium, and escape. Here we present a model for a virus that causes cancer where immunoediting is coordinated through synergistic viral- and host-mediated events. We argue that the initial viral replication process of the Human T cell leukemia virus type I (HTLV-1), which causes adult T cell leukemia/lymphoma (ATL) in ~5% of individuals after decades of latency, harmonizes with the host immune system to create a population of cells destined for malignancy. Furthermore, we explore the possibility for HIV to fit into this model of immunoediting, and propose a non-malignant escape phase for HIV-infected cells that persist beyond equilibrium.

## Introduction

Cancer immunoediting describes the dynamic reciprocity in which the immune system both protects against cancer while inadvertently sculpting a population of cells that may become malignant, progressing through three distinct phases: elimination, equilibrium, and escape [reviewed in (1, 2)]. During elimination, the coordination of innate and adaptive immunosurveillance enables the detection and destruction of early potential tumor cells, while some cells evade the immune response. Cells that survive elimination persist through what can be decades of equilibrium in a dormant state with continued selection pressure that promotes the survival of cells that have developed immunoevasive phenotypes. These cells proceed into the escape phase with acquired somatic mutations and genetic instability that drive their ability to proliferate indefinitely while maintaining invisibility from immunosurveillance, ultimately causing malignancy in the individual (1, 2).

As we discuss what is known about immunoediting in cancer to elucidate the capacity for immunoediting to occur in HIV infection, as discussed in detail in our sister publication in this issue, we explore the intersection of cancer caused by a virus to highlight differences between host- vs. viral-mediated immunoediting, to reveal whether we can untangle the two concepts. Human T cell leukemia virus type 1 (HTLV-1) was the first retrovirus identified to infect humans and was discovered as the etiological agent of adult T cell leukemia/lymphoma (ATL) (3, 4). The prevalence of ATL is as high as 20% among carriers who were born with or contracted HTLV-1 around birth, and infected children have a 25% lifetime risk of developing ATL (5). ATL is extremely aggressive with poor survival outcomes, and even with intensive chemotherapy or allogeneic hematopoietic stem cell transplantation (HSCT), relapse remains high (6). While cancers caused by viruses may undergo what is classically defined as immunoediting, there exists another layer encoded within the viral genome itself to specifically modify how that cell is able to survive or how it can interact with the immune system. During the decades of latency prior to T cell transformation the processes of mutation, clonal selection and expansion, and selection by the immune system allows for HTLV-1 infected cells to complete the immunoediting process in carriers who develop ATL. This is due to the myriad of epigenetic and genetic changes that accumulate over time, initiated early by viral-mediated events that fated this specific cell to eventual transformation.

## Events During Early HTLV-1 Infection set the Stage for the Elimination Phase of Immunoediting

There exist many parallels between HTLV-1 infection and the course of HIV infection, especially in the case of people living with HIV who are treated with suppressive antiretroviral therapy (ART), including their preferential infection of activated memory CD4^+^ T cells (7) followed by integration into transcriptionally active regions within the host genome (8, 9). Unique integration sites arise through the infectious spread of both viruses, with decades of mitotic division resulting in the clonal expansion of infected cells (10–12). In contrast, unlike the exhaustive viral replication of HIV that leads to an average of 10^5^ virions per mL of plasma (13–16), the productive replication of HTLV-1 spreads through the virological synapse (17) in the absence of detectable cell-free virions in peripheral blood (10).

Early in viral replication, the HTLV-1 Tax protein acts as a transcriptional transactivator of the viral long terminal repeat (LTR) (18, 19) analogous to HIV Tat (20). Tax acts through binding host cAMP response element binding protein (CREB) to recruit histone acetyl transferases to the Tax-responsive element 1 (TRE-1) to promote viral transcription (21, 22). Tax can also transcriptionally activate the expression of or alter function of cellular proteins with roles in T cell activation, proliferation, and survival (23–34). Particularly, Tax can inactivate the transactivation function of cellular p53 by inhibiting its N-terminal activation domain (35), abrogating the p53-induced G_1_ cell cycle arrest required to allow appropriate repair in response to DNA damage (36). Tax alters the expression of many host proteins associated with cell cycle, accelerating progression through G_1_ and disabling checkpoints at cell cycle transitions, meanwhile stimulating antiapoptotic signals, and affecting telomerase expression (37). Maintaining the cell in a metabolically active state confers a fitness advantage for viral replication but with grim consequences to the host cell, potentially enabling chromosomal instability, and the accumulation of host genomic mutations (37).

Infectious spread of HTLV-1 establishes thousands of infected-cell clones and then peaks within 3 months of infection before plateauing (38) to a level that is dependent upon the quality of the individual's mounting immunity (39). The proviral genome encodes its own mechanism to impede the activity of Tax protein. The 3′LTR drives an antisense transcript, expressing HTLV-1 basic leucine zipper factor (HBZ) (40) which can outcompete Tax in binding CREB, blocking interaction with TRE-1 and downregulating viral transcription (40, 41). Tax and HBZ rival in many host cell signaling pathways to alter viral replication and change expression profiles within the cell to coordinate proliferation and survival of HTLV-1 infected cells (42). Where Tax activates pathways including NF-kB, AP-1, NFAT, and Wnt signaling, HBZ acts to repress them (30, 32, 43–46). By hindering ongoing viral replication, cells expressing HBZ are driven into a latent state (40).

Although the control of latency is not as well-defined as in HIV, there are specific cellular attributes that repress HTLV-1 expression. DNA methylation accumulates along the provirus after seroconversion and throughout chronic infection, which is not correlated with the methylation patterns of host genes surrounding viral integration sites (47–49). Early methylation is observed in regions that encode Gag, Pol, and Env, with the 5′LTR becoming heavily methylated over time; sometimes associated with hypoacetylated histones, silencing viral sense transcription (47–49). The 3′LTR remains unmethylated, permitting continued expression of HBZ to drive clonal proliferation (47, 48). These distinct patterns of epigenetic modification are established through the 11-zinc finger protein CCCTC-binding factor (CTCF)—a host insulator element that restricts the spread of epigenetic modifications to define boundaries between transcriptionally active and inactive regions of the genome (50, 51). The binding of CTCF to proviral DNA at the defined epigenetic border modifies viral transcription and splicing (50), contributing to the regulation of latency as similarly observed in EBV (52) and KSHV (53).

Virus-coordinated events early in replication alter the population of CD4^+^ T cells infected with HTLV-1. Whether Tax expression is a prerequisite for malignancy remains debated in the field, yet we theorize that initial Tax activity is a major driver of immunoediting. Tax-induced changes to the cell that promote viral protein expression and the presentation of neoantigens provoke immunosurveillance and progression into the elimination phase of immunoediting. Initial Tax activity changes the cell and may predestine it to become malignant (31, 34), should it survive the robust HTLV-1 specific immune response and acquisition of appropriate somatic mutations through decades of latency.

## Elimination: How the Host Immune Response Sculpts the Persistence of HTLV-1 Infected Clones

The interplay between early viral protein expression and the establishment of HTLV-1 latency synchronize with host immunosurveillance into the elimination phase of immunoediting, where a strong immune response remains unable to eradicate the virus and does not intrinsically prevent ATL (54). CD8^+^ Cytotoxic T lymphocyte (CTL) responses are detected against viral Gag, Pol, and Env, although Tax remains the immunodominant target (55–61). Studies in rats demonstrate that siRNAs against Tax sufficiently downregulate Tax expression, repressing Tax-specific CTL killing of HTLV-1 infected cells (62). In asymptomatic individuals, whilst Tax expression remains low or undetectable immediately *ex vivo*, short-term culture of CD4^+^ T cells is sufficient to reactivate viral expression from latency which is rapidly controlled with the addition of autologous CD8^+^ T cells (61). There exists sequence heterogeneity across HTLV-1 isolates, although not as extensive as the diversity observed in HIV that drives the emergence of CTL escape mutants (63, 64). Natural variation in the *tax* gene, however, can lead to peptide presentation that cannot be recognized by consensus-sequence Tax-specific CTLs (63). These variants render severe functional impairment of Tax activity, and therefore a survival advantage that enables the maintenance of a population of cells with reduced sense transcriptional activity that continue to evade immune recognition (59, 63).

Chronically active HTLV-1 specific CTLs are present in otherwise asymptomatic carriers of HTLV-1 without associated disease (58), and the proliferation rates of memory CD8^+^ T cells are 3-fold higher than in uninfected controls (65). The frequency of HTLV-1 specific CTLs does not correlate with proviral loads, while transcriptomic analysis of CD8^+^ T cells reveals that individuals with low proviral loads highly express gene clusters associated with improved effector function, and with CTL-mediated lysis (66). Additionally, Tax-expressing CD4^+^ T cells increase the expression of molecules, i.e., ICAM-1, Fas, and TRAIL-R1/2, improving the susceptibility of these cells to CTL-mediated lysis (60). These data support the notion that bursts of antigenic stimulus throughout latency drive persistent immunosurveillance and depletion of infected cells expressing antigen, suggesting an equilibrium is established between replicating virus and the immune response (58, 59, 66, 67).

The infected individual's human leukocyte antigen (HLA) alleles restrict the repertoire of antigen presented to CTLs (60, 68). HLA-A^*^02 binds various Tax epitopes, with a particularly strong affinity of Tax_11−19_ for the peptide binding groove of A^*^02, which confers a lower proviral load and selective pressure against Tax-expressing cells in asymptomatic carriers (54, 58, 69–71). HBZ also binds to the protective alleles A^*^02 and CW^*^0801, leading to lower proviral loads in asymptomatic carriers (54). However, the frequency of HBZ-specific CTLs is significantly lower than Tax-specific CTLs, and the binding affinity of HBZ to HLA molecules is notably weaker than Tax peptides (54, 60). The low immunogenicity of HBZ in asymptomatic carriers is mirrored in subsequent malignancy—ATL cells constitutively express HBZ, yet HBZ-specific CTLs fail to lyse transformed ATL cells (72). However, more recent work has demonstrated that vaccines harboring specific HBZ epitopes, i.e., HBZ_157−176_, can elicit anti-HBZ CTLs in model ATL mice (73), warranting further research to improve the immunogenicity of low-affinity HLA-associated HBZ peptides to enhance ATL immunotherapy (74). That HBZ does not stimulate strong CTL responses may be surprising, given its imperative action in maintaining the survival and proliferation of infected cells throughout all phases of immunoediting. This could be due to the additional function elicited by *HBZ* in its RNA form, inducing distinct antiapoptotic activity, therefore precluding the expression of antigen while promoting cell survival (75).

The capacity of HBZ to downregulate Tax-induced viral transcription occurs, in part, to evade the stronger immunodominant Tax-specific CTLs. The dynamic coordination between early virus- and immune-mediated events permit the sufficient control of active viral replication while creating longevity in a population of infected cells refractory to immune recognition, inherently sculpting the persistence of certain clonal populations of infected CD4^+^ T cells that do not express Tax. This elimination phase of immunoediting synchronized by the virus and the host immune response orchestrates the immortalization of infected cells without transforming them, securing their persistence for years.

HIV also evades immune recognition, albeit through different mechanisms including Nef-directed downregulation of CD4 and MHC class I receptors (76, 77). In our sister article, we describe in depth, models of immunoediting during HIV infection and the progression to AIDS, as well as through suppressive ART. Entry into latency is established soon after HIV transmission, either by infected activated CD4^+^ T cells reverting back to a resting state (78–81) or through the direct infection of resting CD4^+^ T cells (82–85). Individuals living with HIV who have access to ART can achieve undetectable viral loads (13–16). This medical intervention (which is not required to inhibit the infectious spread of HTLV-1) allows for the recovery of CD4^+^ T cells (13–16). Consequently, this recovery enables clonally infected populations that exist before the initiation of ART (86) or that are seeded as viral replication wanes, to expand and contract (87) through years of equilibrium congruent with this model of immunoediting.

## Equilibrium: Viral and Host Factors that Contribute to the Continued Selection and Survival of HTLV-1 Infected Clones

**Figure 1:**
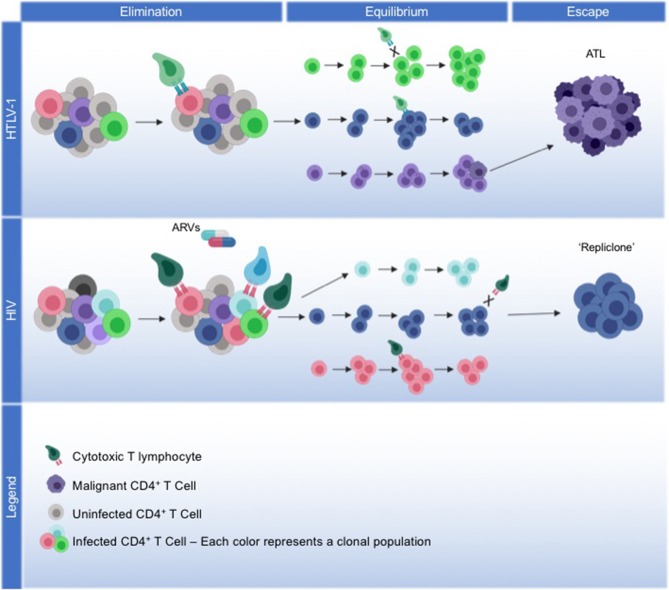
Model for viral and host coordinated immunoediting. **HTLV-1**: We propose a model of immunoediting that leads to ATL, mediated through HTLV-1 replication, and the host immune response. HTLV-1 infection spreads through the virological synapse without cell-free virions, maintaining low proviral loads. The viral protein Tax drives viral replication and alters the expression or activity of many host proteins involved in survival and proliferation, facilitating the immortalization of infected cells. During the elimination phase, the development of a robust Tax-specific CTL response will kill off cells that continue to express Tax, and infection plateaus. HBZ expression from the antisense transcript will repress viral transcription to protect cells from CTL killing, meanwhile driving them into latency. Immortalized cells that can evade the immune response are selected into equilibrium, where continued HBZ expression maintains the survival and proliferation of latently infected cells. Over decades of equilibrium, clonal populations with identical proviral integration sites continue to proliferate, accumulating somatic mutations and epigenetic modifications that may lead to the eventual transformation into ATL. Through the escape phase, a malignant cell emerges from a monoclonal population, with HBZ expression and the acquired somatic mutations enabling ATL cells to continue to proliferate and evade cancer immunosurveillance. **HIV**: We propose that HIV, in part, fits into this immunoediting model, albeit with differing mechanism. During the elimination phase, robust viral replication of HIV initially establishes high proviral load, with strong HIV-specific CTL responses enabling infection to plateau. With the addition of antiretrovirals (ARVs), infection is driven into latency and as viral replication ceases, latently-infected cells not recognized by CTLs will persist into the equilibrium phase. Throughout decades of latency, cells with the same proviral integration sites will clonally expand, with clonal populations waxing and waning over time. Less is known about what drives the proliferation of certain HIV infected clonal populations. Recently, the concept of a “repliclone” has been established, representing the expansion of a monoclonal population with replication competent provirus defined by a single integration site. Although these cells are not malignant, they persist into the escape phase given a yet unknown selection advantage for survival and proliferation.

HTLV-1 infected cells that have survived the elimination phase, and exist in latency with an immune-evading phenotype, will endure into the equilibrium phase of immunoediting. During equilibrium, sustained polyclonal expansion of infected cells will favor clonal populations that continue to accumulate somatic changes that facilitate cell survival (Figure 1).

HTLV-1 integrates preferentially into transcriptionally active regions of the host genome (88, 89), with a modest preference for integration near host transcriptional start sites (90), with a small percentage of ATL cases (<6%) containing proviruses near genes associated with hematological malignancies (91). Asymptomatic carriers harbor between 10^4^ and 10^5^ clones with unique integration sites (92, 93) capable of indefinite proliferation (51) and a preference for the expansion of clones containing proviral integration within the long arm of acrocentric chromosomes 13, 14, 15, and 21 (91). The hypothesized survival of these particular integration sites is that these chromosomes are physically associated with the nucleolus of non-dividing cells, and this nucleolar periphery remains transcriptional quiescent—such that these cells evade HTLV-1 specific CTL killing (51, 91).

Throughout decades of equilibrium, clonal populations are selected for, expand and contract, and integration site sequencing reveals that 90% of cancer cells from patients with ATL are expanded from a single predominant malignant clone (91, 94). What drives the selection of this monoclonal population of malignant cells in the background of polyclonal populations that fail to transform? The prevalence of each clonal population represented by the proportion of observed integration sites to PVL suggests that a malignant clone emerges from an initially low-abundant clonal population (91), rather than a largely expanded clonal population (11, 93). While high-abundance clones manage to progressively increase over time, low-abundance clones tend to decay, likely due to their modest levels of Tax expression during latency (51, 93). Low-abundance clones that progress to ATL display the highest level of integration within acrocentric chromosomes 13 and 15 (91), potentially contributing to sporadic Tax expression during cell division- or stress-induced dispersal of nucleoli, temporarily releasing viral transcriptional repression (51). There are documented plus-strand transcriptional bursts during chronic infection that enable intermittent expression of Tax protein (95) that can trigger antiapoptotic machinery (96), thought to contribute to survival of infected cells and lasting Tax-specific CTL responses (51) and an additional push toward transformation into ATL cells.

Each clone has a particular susceptibility to experience malignant transformation, initially predisposed through Tax-induced changes to the cell (51), as described above. While Tax is not commonly expressed in latency or transformed ATL cells, HBZ is constitutively expressed throughout chronic infection and in ATL, and displays oncogenic properties (51, 97). HBZ promotes proliferation by targeting retinoblastoma tumor suppressor protein (98) and further inhibits apoptosis by repressing the transcriptional activity of p53 (99, 100), and by suppressing the pro-apoptotic genes *Bim* and *Fas ligand* by downregulating their transcriptional activator, FoxO3a (101). Even in the absence of Tax expression, HBZ can maintain the immortalized status of the cell and continues to drive clonal expansion.

Proliferation of these clones over decades also allows for the accumulation of somatic mutations due to random errors during DNA replication, i.e., somatic mutations (102). Independent of Tax- or HBZ-induced inhibition of p53 function, which occurs in the presence of wild type p53 protein, 30–40% of ATL patients have acquired mutations in the p53 gene (23, 103–105). In an incredible integrated molecular analysis of ATL cells from 426 individuals, Kataoka et al. investigated the whole-genome exome, transcriptome, and methylome of ATL cells (97). They identified significant mutations in 50 genes, with over 30% of mutations observed in both the phospholipase C γ1 (PLCγ1) gene and a member of the PKC family of proteins (PRKCB), additionally correlated to mutations in the cytoplasmic scaffolding gene *CARD11*, with RNA sequencing confirming transcripts with acquired mutations (97). Other hotspot mutations were observed in genes within the same pathway, and although there were no functional analyses on the acquired mutations in this study, literature in other cancers indicate that together the observed changes in amino acid sequence are gain-of-function mutations in this set of genes, and can act to increase TCR signaling and antigen-receptor induced NF-kB activation of T cells (97). Interestingly, 56% of ATL cells exhibited deleterious mutations that would predispose them to evade immunosurveillance, including within the major histocompatibility complex (MHC) class I, immune checkpoints, and death signaling pathways. The MHC class I gene was also discovered to be extensively hypermethylated, with 90% of ATL cases harboring mutations and/or methylation patterns within this gene that would render a loss of expression of MHC class I (97). Other mutated pathways discovered through this study are common in other human malignancy, including DNA repair mechanisms, epigenetic regulation, and telomere preservation. Overall, the accumulation of these mutations over time demonstrate the ability of cells to continue to proliferate while evading immune response.

In addition to the integration site and accumulation of somatic changes, another mechanism for persistence is the accumulation of clonal populations containing defective provirus. Defective proviral genomes may lack the 5′LTR and flanking genomic regions that encode immunogenic gene products, particularly Tax, contributing to immune evasion (106, 107). Defective proviral genomes that explicitly express HBZ can proliferate and avoid immunosurveillance, while driving cells toward malignancy. HBZ is sufficient to stimulate T cell lymphoma in mice in the absence of any other viral proteins (108) even after a period of latency (109), and the concept that HBZ alone could induce ATL has been observed *in vivo* (107). While HBZ is ubiquitously expressed in ATL (97), defective proviruses are observed in up to 56% of ATL cells (107, 110, 111). Tamiya et al. identified the existence of what they termed a type II defective provirus as one that contains a large recombination between *env* and the 5′LTR, partially deleting the LTR and most of the genome, whilst others have identified the complete deletion of the 5′LTR (112, 113)—in both cases, only an active 3′LTR remains, and individuals with type II defective proviruses have the most aggressive forms of ATL (107). Cells that harbor defects in the 5′LTR that preclude Tax expression would completely evade Tax-specific CTLs. The discovery of these type II defective proviruses was made over a decade before the identification of HBZ; Tamiya et al. could only speculate at this point that a viral gene other than *tax* could potentially drive transformation, and they were correct. It is now widely accepted that an intact 3′LTR and *HBZ* gene are essential for oncogenesis (51, 113, 114).

Further supporting Tax immortalization but HBZ oncogenesis is the closely related retrovirus, HTLV-2, which is not associated with malignancy (115, 116). The Tax protein of HTLV-2 has demonstrated behavior in driving T cell immortalization by promoting survival and abnormal proliferation through similar mechanisms observed by HTLV-1 Tax (117). HTLV-2 also encodes an antisense protein, APH-2, which can repress HTLV-2 Tax-mediated transcription (118, 119), yet does not exhibit the oncogenic properties of HBZ, and does not promote malignancy (120). These studies suggest the oncogenic behavior of HBZ is distinct from Tax-driven immortalization of CD4^+^ T cells.

HTLV-1 infected clonal populations are selected for through decades of equilibrium from the initial immortalization of these cells by Tax, with maintained survival achieved from the ubiquitous expression of HBZ. In addition, certain common integration sites, proviral defects, and the acquisition of genetic and epigenetic changes that drive proliferation whilst protecting against immune responses promote further survival of these clones. While HIV latently-infected cells with identical integration sites are also demonstrated to undergo clonal proliferation (9, 12, 121, 122), it remains unknown what drives this expansion. Similar to HTLV-1, HIV preferentially integrates within introns of actively transcribing genes. And while HIV does not express a protein analogous to HBZ to promote cell proliferation, HIV has demonstrated integration patterns within genes responsible for controlling cell division and growth, perhaps contributing to their clonal expansion (12). Despite different mechanisms, both retroviruses promote the clonal proliferation of their latently infected cells. HTLV-1 infected CD4^+^ T cells expand in a polyclonal manner, with a dominant clone selected for malignancy in a fraction of individuals (Figure 1). Although HIV does not transform cells, polyclonal populations wax and wane throughout latency (87), raising the possibility that particular populations could be selected for through the escape phase of immunoediting.

## Escape: How a Single HTLV-1 Clone Becomes Cancer

Now that HTLV-1 infected cells have survived through decades of equilibrium, a select monoclonal population that has accumulated the appropriate set of mutations, and which remains resistant to immune defenses, will enter the escape phase of immunoediting, and become the malignant population of ATL cells in 5 to 20% of individuals living with HTLV-1. ATL is classified into four clinical subtypes, acute, lymphoma, chronic, and smoldering; each with varying clinical manifestations, pathogenesis, and treatment strategy (123). Cells that have undergone transformation into ATL display hallmarks of malignancy, the majority of which are driven by HBZ activity—some identified hallmarks have enabled the development of new targeted therapies for ATL.

Now that an HTLV-1 infected clonal population has become malignant, it must continue to counteract cancer-specific immunosurveillance, not only HTLV-1-specific immune responses. A variety of cancers can manipulate immune checkpoint expression to evade immunosurveillance, leading to the development of immune checkpoint blockade as a successful therapeutic strategy for certain cancers (124). The co-inhibitory receptor T cell immunoglobulin and ITIM domain (TIGIT) is a well-characterized inhibitory checkpoint. When upregulated on CD4^+^Foxp3^+^ T cells, TIGIT induces the expression of IL-10, contributing to dysfunctional CD8^+^ T cells within tumor microenvironments (125). Classically, CD4^+^Foxp3^+^ T cells are defined as T regulatory (Treg) cells, and up to 70% of transformed ATL cells express Foxp3 (126–130). These findings initially associated ATL with Tregs, even suggesting that ATL originates from the Treg subset infected by HTLV-1 (131). More recent findings have demonstrated that HBZ can modify the immunophenotype of conventional CD4^+^ T cells to exploit the desired properties of Treg cells while impairing their suppressive function (109). HBZ can enhance *Foxp3* transcription and hijack its function to stimulate proliferation of ATL cells (44, 109, 132). TIGIT is also highly expressed on ATL cells (132–134). HBZ directly increases the expression of TIGIT to protect ATL cells from immunosurveillance (132), meanwhile abrogating the inhibitory effect TIGIT generally has on T cell proliferation, allowing the cells to continue to proliferate (133). HBZ can also induce the expression of the immunosuppressive cytokine IL-10, increasing its secretion from ATL cells, further supporting the role HBZ plays in the evasion of anti-viral and anti-cancer host defenses (132).

Expression of the host CC chemokine receptor CCR4 is observed in 90% of tumor cells isolated from individuals living with ATL (135–137). Gain-of-function mutations in the *CCR4* gene enhance the chemotactic properties of ATL cells, thought to drive infiltration into organs by impairing CCR4 internalization, and improve cellular metabolism and survival by prolonging PI3K/AKT signaling (97, 138). HBZ can induce the expression of CCR4 through its major transcription factor (GATA3), driving migration and proliferation of ATL cells (139). These findings led to the development of mogamulizumab, a defucosylated monoclonal antibody against CCR4, which was recently approved for treatment of relapsed ATL (137, 140, 141). The defucosylated portion of the Fc region of mogamulizumab enhances antibody-dependent cellular cytotoxicity (ADCC) against CCR4^+^ ATL cells given an increased affinity to bind the Fc receptor on effectors cells (137, 142, 143). In a small clinical study to investigate the dynamics of this successful monotherapy, mogamulizumab was demonstrated to reduce proviral load, with a particularly rapid reduction of the abundance of the CCR4^+^ malignant clone (144). Researchers suggest that individuals with high levels of CCR4^+^ HTLV-1^+^ cells could benefit from this therapy to prevent the development of ATL. This type of immunotherapy, however, must proceed with caution. CCR4 is expressed on Tregs to drive their migration to and mitigate inflammatory responses in tissue (145), and while a reduction in Tregs may boost immunity in the tumor microenvironment, it may simultaneously create autoimmunity in tissue sanctuaries (146). Although mogamulizumab treatment is demonstrated to maintain ATL remission and decreased HTLV-1 proviral loads, the associated reduction in normally functioning Tregs can cause severe adverse events, as observed in individuals with ATL treated with mogamulizumab; one individual developed fatal Stevens-Johnson syndrome due to reduced Treg populations (146).

Overall, immunoediting coordinated by the virus and by the host immune response has inherently compelled particular HLTV-1 infected cells down a pathway toward cancer. This transpires in at least 5% of individuals, and within those individuals, only occurs from 1 in 10^5^ unique clones. ATL is rare, yet very aggressive. It takes 40–50 years for infected cells to transform and depends on the initial immortalization of cells by Tax, years of proliferation driven by HBZ, and the accumulation of the appropriate set of host cell genetic and epigenetic changes, all while evading HTLV-1- and cancer-specific immunosurveillance. In this circumstance, immunoediting has enabled the immune system to protect against viral infection whilst promoting the persistence of cells fated to become malignant, and the historical evolution of HTLV-1 with human cellular machinery has created a symbiotic relationship between the virus and its host cell, at eventual the cost of human lives.

## What Does HIV Look Like in the Escape Phase Under This Model?

There have been no reported cases to date of HIV causing malignancy in CD4^+^ T cells, which is likely due to the fact HIV does not express a protein homologous to HTLV-1 HBZ, the driver of ATL. Although HIV and HTLV-2 both encode an antisense element [ASP (147) and APH-2 (118), respectively], neither display oncogenic properties (120, 148). What does the escape phase of immunoediting look like for HIV to fit into this model, even without malignancy? We propose that the final “escape” of clonally expanding cells that remain refractory to immunosurveillance are those recently termed “repliclones,” an emerging concept in which CD4^+^ T cells harbor full-length replication-competent proviral sequences with identical integration sites, sometimes contributing to clinically-detectable viremia in individuals on long-term suppressive therapy. Although these integration sites represent rare events in the background of the multitude of defective proviral genomes, the clones themselves are large, with an estimated expansion between 50 to 300 million CD4^+^ T cells (Halvas EK; Conference on Retroviruses and Opportunistic Infections 2019; Seattle, Washington) (Figure 1). Currently, there is no evidence that they are selected for, and research is warranted to define mechanisms that drive their capacity to expand and potentially contract, and to elucidate their ability to produce clinical viremia in the presence of circulating HIV-specific CD8^+^ T cells.

There are multiple suggested and demonstrated mechanisms that drive the clonal expansion of HIV latently infected cells, encompassing progression through normal T cell function undergoing homeostatic proliferation while enduring bursts of expansion in response to antigen (149). In a case study, Simonetti et al. reported on an individual that developed low-level viremia after 12 years on ART. Single genome sequencing of plasma viral RNA revealed a portion of this population was genetically diverse and viremia was attributable to drug resistance. The other portion of viral RNA was identical, and lacked drug resistance mutations. Changing ART regiment suppressed the diverse sequences with resistance mutations, but did not affect the identical sequences. These sequences were mapped to an integration site in an ambiguous region of the human genome, thus labeled AMBI-1, with an estimated expansion to 9 million cells over time (150). The authors suggest that this clone could persist and expand over years whilst producing virus particles because this individual never achieved full T cell recovery, potentially impairing immune responses. Additionally, this individual developed squamous cell carcinoma, and AMBI-1 RNA sequenced from plasma waned post-cancer treatment, but reemerged with its relapse. Autopsy of the metastatic lesions revealed that infiltrating CD4^+^ T cells in tumor tissue were enriched for AMBI-1 clones (150). This suggests proliferation of this clone could have been driven through response to tumor antigen (12), and is consistent with a model where response to antigens or homeostatic proliferation are the major drivers of clonal expansion. In some cases, the proviral integration site itself may also contribute to enhanced clonal expansion and persistence of cells. This is supported by studies which have reported over-representation of expanded clones with integrations into genes associated with proliferation, i.e., *MKL2* and *BACH2* (12). The study of factors responsible for driving clonal expansion in HIV remains an active and important area of study.

Although the repliclone does not become malignant as observed in HTLV-1, it follows the concept that a clonal population can be selected for, refractory to immune response, and can survive for years. Given these expanded clones are difficult to study, evidence for them is limited, with more reports emerging at conferences. It appears that the escape of a repliclone has many mechanisms at play and will likely differ across individuals. Whatever the mechanism of expansion, it remains clear that these repliclones do persist, and perhaps have accumulated survival phenotypes throughout decades of elimination, antiretroviral therapy, equilibrium, and escape. As discussed in our partner manuscript, we have recently described that replication competent HIV-infected cells from individuals on suppressive ART are resistant to HIV-specific CTL killing, even when stimulated to reactivate latent infection (151). The inherent survival of these cells that have undergone robust T cell activation and are otherwise hardwired to achieve successful viral replication and antigen presentation with susceptibility to CTL killing, suggests these cells have acquired a unique feature or set of features through the various phases of immunoediting that facilitate proliferation and survival and the acquisition of an immune-evading phenotype. Research to uncover these mechanisms of persistence and immune evasion is extremely relevant for the field, and may elucidate treatment strategies to curb populations that otherwise continue to expand and greatly contribute to the perseverance of the stable, replication competent latent reservoir.

## Author Contributions

All authors listed have made a substantial, direct and intellectual contribution to the work, and approved it for publication.

### Conflict of Interest

RJ declares that he is a member of the AbbVie Inc. Scientific Advisory Board, and has received payment for this role. The remaining author declares that the research was conducted in the absence of any commercial or financial relationships that could be construed as a potential conflict of interest.

## Abbreviations

HTLV-1: human T cell leukemia virus type I
ATL: adult T cell leukemia/lymphoma
CTL: cytotoxic T lymphocyte
HIV: human immunodeficiency virus
ART: antiretroviral therapy.
